# Disparities in diagnosis and outcomes in American patients with transthyretin cardiac amyloidosis

**DOI:** 10.1007/s10554-025-03436-4

**Published:** 2025-06-05

**Authors:** Joyce L. Ngouchet Nouhossi, Iva Minga, Teodora Szasz, Vien T. Truong, Amber E. Johnson, Edward Yang, Srisha Kotlo, Varun Subashchandran, Frank Medina, Karolina M. Zareba, Akash Goyal, Orlando P. Simonetti, Amit R. Patel, Cristiane C. Singulane, Jai Singh, Vidya Nadig, Shaimaa Fadl, Cory R. Trankle, Nitasha Sarswat, Hena N. Patel, Victor Mor-Avi, Bryan Smith, Jeremy A. Slivnick

**Affiliations:** 1 University of Chicago Medicine, 5758 S. Maryland Ave, MC 9067, Chicago, IL 60637 USA; 2 Philips Healthcare, Cambridge, MA USA; 3 Nazareth Hospital, Philadelphia, PA USA; 4 The Ohio State University Wexner Medical Center, Columbus, OH USA; 5 University of Virginia, Charlottesville, VA USA; 6 Atrium Health, Charlotte, NC USA; 7 Hartford Hospital, Hartford, CT USA; 8 Virginia Commonwealth University, Richmond, VA USA

**Keywords:** Cardiac amyloidosis, Ethnic disparities, Cardiac magnetic resonance, Heart failure, Cardiomyopathy

## Abstract

Although Afro-Caribbean (AC) race has been associated with worse outcomes in many cardiovascular diseases, its potential association with transthyretin cardiac amyloidosis (ATTR-CA) is less understood. We aimed to assess the relationship between race and serum biomarkers, adverse cardiac remodeling, and outcomes in AC vs white ATTR-CA patients. 114 AC and 117 white patients confirmed ATTR-CA who underwent cardiac magnetic resonance (CMR) exam were identified. The relationship between race and the primary endpoint—defined by all-cause mortality or heart failure hospitalization—was assessed using Cox regression analysis. ATTR disease stage was significantly higher at diagnosis in AC vs white patients (p < 0.0001). Left (p = 0.001) and right ventricular ejection fractions (p = 0.0002) were lower and extracellular volume (58% vs 50%) higher in AC vs white patients. At a median follow up time of 365 (IQR, 97–879) days, 44% of patients had experienced the primary endpoint. AC race was strongly associated with the primary endpoint compared with White patients (HR 2.83, 95% CI 1.92–4.23, p < 0.0001). AC patients were found to be at more advanced disease stages at the time of ATTR-CA diagnosis and experienced poor outcomes more frequently, highlighting the need for targeted strategies to address these health inequities.

**Supplementary Information:**

The online version contains supplementary material available at 10.1007/s10554-025-03436-4.

## Introduction

Cardiomyopathy due to amyloid depositions occurs when misfolded proteins—typically transthyretin or immunoglobulin light chains—accumulate in the myocardium, potentially resulting in heart failure, arrhythmias, and sudden death. Transthyretin amyloid cardiomyopathy (ATTR-CA) is an increasingly recognized and treatable cause of heart failure [1, 2]. ATTR-CA may occur either spontaneously as a wild type (ATTRwt-CA) or in the context of a pathogenic gene mutation (ATTRv-CA) [3]. As compared to ATTRwt-CA, ATTRv-CA typically manifests earlier with variable degrees of neurologic and cardiac involvement, depending on the specific mutation [3]. Although a majority of ATTRwt patients are white, certain ATTRv gene mutations—particularly Val142Ile—affect predominantly Afro-Caribbean (AC) populations [4]. Irrespective of ATTR genotype, delays in diagnosis are common in this disorder and may attenuate the benefits of life prolonging therapies such as tafamidis [1, 5]. Therefore, it is imperative that ATTR-CA be diagnosed and treated as early as possible, prior to irreversible cardiac damage.

Prior studies have shown that racial disparities exist in diagnosis and prognosis in the heart failure population. Multiple large population studies have demonstrated increased incidence of heart failure and related hospitalizations amongst AC patients, as compared to white patients [6–8]. Additionally, studies have shown that patient race may negatively impact decision-making regarding the pursuit of life saving therapies, such as implantable cardiac defibrillators and advanced heart failure therapies [9–12]. Similar disparities have been identified with respect to race in other cardiomyopathies [13, 14]. Although the impact of race on cardiac remodeling and outcomes has been described in the HF population at large, its potential significance specifically in ATTR-CA is poorly understood.

Given the relationship between race and adverse outcomes in other cardiovascular disorders, we hypothesized that AC race would be similarly associated with more advanced disease at the time of diagnosis, reduced treatment rates, and worse outcomes in ATTR-CA. The impact of such findings would be of critical clinical significance given the racial predominance of ATTR-CA in AC patients. Accordingly, in this large, multicenter study, we aimed to assess the potential impact between AC race and these factors in ATTR-CA, using clinical and cardiac magnetic resonance-based (CMR) biomarkers.

## Methods

### Study population

Using the Society of Cardiovascular Magnetic Resonance (SCMR) Registry, we retrospectively identified 231 patients with confirmed ATTR-CA diagnosis who underwent CMR examinations at four academic centers between 2007 and 2023 (Atrium Health, The Ohio State University, University of Chicago, University of Virginia). Patients were identified by each participating site, and all eligible patients with confirmed ATTR-CA scanned during this timeframe were included. The SCMR registry is a multicenter, collaborative, international repository of de-identified CMR reports and Digital Imaging and Communications in Medicine images [15]. A diagnosis of ATTR-CA was made according to current guidelines using positive endomyocardial biopsy, positive extracardiac biopsy with typical cardiac imaging features, or a grade ≥ 2 uptake on technetium pyrophosphate (Tc-PyP) scan in the absence of a monoclonal light chain protein on comprehensive serum and urine analysis [16]. Time of diagnosis was defined based on the date of positive Tc-PyP or invasive tissue biopsy. 6 patients were neither white nor AC were excluded from the study. This study was approved by the institutional review s of each participating institution with a waiver of informed consent.

### Clinical data and lab biomarkers

Demographic variables at the time of CMR exam, including age, sex, comorbidities such as a history of hypertension, coronary artery disease, hyperlipidemia, diabetes, and New York Heart Association (NYHA) heart failure class, were extracted from electronic medical records. Self-identified race was obtained from the electronic medical record. Serum creatinine, estimated glomerular filtration rate (eGFR), n-terminal BNP (NT-proBNP), and high sensitivity troponin levels at the time of CMR were also abstracted. ATTR-CA stage was calculated according to the United Kingdom Staging Guidelines [17]. The primary outcome of all-cause mortality or heart failure hospitalization (HFH)—defined as admission to the hospital for the diagnosis of heart failure with evidence of congestion requiring intravenous loop diuretics—was obtained through chart review and review of the Social Security Death Index [18]. All-cause mortality and HFH were also considered individually as secondary endpoints.

### CMR image acquisition and analysis

CMR scans closets to the time of diagnosis were used for this study. Cardiac magnetic resonance imaging protocols were performed either at 1.5 or 3.0 T using Philips (Achieva), General Electric (SIGNA Artist AIR edition), or Siemens (Magnetom Avanto, Manetom Espree) scanners. Cine images—including apical 2-, 3-, and 4-chamber and serial short-axis slices—were acquired using steady state free precession imaging. Late gadolinium enhancement (LGE) imaging was performed 8–15 min following administration of a standard dose of gadolinium-based contrast agent in accordance with CMR guidelines [19]. Inversion time was determined using Look-Locker sequence to identify the timing of optimal myocardial nulling [20]. LGE imaging was performed using a gradient-echo inversion recovery sequence with magnitude and phase-sensitive inversion recovery reconstruction [21]. T1 mapping was acquired using a Modified Look-Locker Inversion Recovery sequence with pulse sequence variations based on scanner [22, 23].

Extracellular volume (ECV) was quantified using the patient’s hematocrit and T1 values from the middle third of the mid-myocardial interventricular septum using previously defined methods [24]. Ventricular size, mass, and systolic function were quantified in accordance with CMR guidelines using commercial software (cvi42, Circle Cardiovascular Imaging Inc., Calgary, Alberta, Canada) [25]. Ventricular and atrial volumes—derived using biplane area-length technique—were indexed to body surface area. Maximal end-diastolic wall thickness was measured in cine short-axis view [26]. The presence of LGE was assessed by a trained level-3 CMR reader blinded to the clinical data. If present, LGE was categorized as typical or atypical using previously defined criteria: a typical LGE pattern was defined as abnormal gadolinium kinetics plus the presence of subendocardial or transmural LGE within hypertrophied myocardium, with either circumferential involvement or affecting ≥ 6 myocardial segments [27]. The typical Look-Locker pattern for cardiac amyloidosis was identified as myocardial nulling occurring either before or within 2 slices of blood pool nulling on the TI scout sequence. Abnormal gadolinium kinetics were considered due to their high specificity for cardiac amyloidosis (CA) [28, 29].

### Statistical analysis

Categorical variables are displayed as n(%). Normality was assessed using the Shapiro–Wilk test. Normally and non-normally distributed variables are displayed as mean ± SD and median with interquartile range (IQR), respectively. Clinical, CMR, and outcomes data were compared between AC and white patients using ANOVA, t-test, or Wilcoxon Rank test, as appropriate. Time to the primary and secondary endpoints was compared between AC and white patients using Kaplan–Meier test, with significance assessed using the log-rank test. The relationships between both race and clinical variables and study endpoints were assessed using Cox regression models. A multivariable Cox proportional hazards model, adjusting for sex, hypertension, ATTR genotype, coronary artery disease (CAD), and diabetes, was employed to evaluate the association between race and clinical outcomes. The proportional hazards assumption was satisfied as evidenced by Schoenfeld residuals exhibiting independence from time. Deviance residuals and dfbeta values revealed no significant influential observations. Additionally, considering that deceased patients cannot undergo HF re-hospitalization, a competing risk survival analysis using the Fine and Gray proportional subdistribution hazards model was conducted as a sensitivity analysis to explore the association between race and hospitalization. The relationship between race and the primary endpoint was also assessed within the subgroups of patients who did and did not carry pathogenic ATTR gene mutations.

Furthermore, we employed a multivariable machine learning Recursive Feature Elimination (RFE) method using logistic regression to rank the importance of the clinical variables for predicting time to the incidence of the primary endpoint of death or HFH [30]. In the multivariable RFE process, starting with the full set of variables, at each iteration, the least important variable (as determined by the model coefficients) is eliminated from the current set. This step was repeated until only the pre-specified number of features was left. In our implementation, we ranked all variables from most to least important, thereby allowing us to observe the relative importance of each feature in the dataset.

Hazard ratios are presented as mean and 95% confidence. A two-sided P-value of < 0.05 was considered significant. The statistical analyses were performed using JMP®, Version 17.2.0. (SAS Institute Inc., Cary, NC) and R software, version 3.6.1 (The R Foundation, Vienna, Austria).

## Results

### Baseline demographics

The median age of the cohort was 77 (IQR 71–82) years, and 82% of the patients were male (Table 1). The most common co-morbidities in the cohort included hypertension (69%), diabetes (65%), hyperlipidemia (65%), and CAD (30%). Most patients were diagnosed with NYHA class II and III heart failure (47 and 39%, respectively). Roughly half of patients identified as either AC or white (49 and 51%, respectively).

**Table 1:** Comparison of clinical data and outcomes between AC and white patients

	Entire cohort (n = 231)	AC (n = 114)	White (n = 117)	P value
Clinical parameters				
Age	77 (71–82)	77 (70–82)	78 (73–82)	0.31
Sex n(% Male)	189 (82%)	83 (71%)	106 (93%)	** < 0.0001**
Genotype Positive n(%)	91 (49%)	76 (74%)	15 (18%)	** < 0.0001**
Hypertension n(%)	159 (69%)	93 (79%)	66 (58%)	**0.0004**
Diabetes n(% Yes)	64 (27%)	41 (35%)	23 (20%)	**0.01**
CAD n(% Yes)	70 (30%)	27 (23%)	43 (38%)	**0.02**
Hyperlipidemia n(%)	104 (65%)	47 (59%)	57 (72%)	0.08
NYHA class				
I	15 (8%)	5 (5%)	10 (11%)	** < 0.0001**
II	85 (47%)	30 (33%)	55 (62%)	
III	71 (39%)	49 (54%)	22 (25%)	
IV	9 (5%)	7 (8%)	2 (2%)	
Mayo stage				
I	78 (35%)	24 (21%)	54 (50%)	** < 0.0001**
II	102 (46%)	61 (53%)	41 (38%)	
III	44 (20%)	30 (26%)	14 (13%)	
NT-proBNP (pg/mL)	2,462 (981–5521)	4319 (1262–7337)	1457 (624–3420)	**0.0002**
Hs-Troponin (ng/mL)	67 (36–111)	78 (57–140)	53 (30–102)	**0.01**
EGFR	59 (45–71)	53 (42–67)	63 (51–73)	**0.002**
Outcomes				
ATTR treatment n(%)	129 (81%)	58 (68%)	71 (95%)	** < 0.0001**
Death n(%)	79 (34%)	53 (46%)	26 (23%)	**0.0002**
HFH n(%)	99 (44%)	75 (65%)	24 (22%)	** < 0.0001**
Death or HFH n(%)	122 (53%)	84 (72%)	38 (33%)	** < 0.0001**

*AC* Afro-Caribbean, *BMI* body mass index, *HTN* hypertension, *DM* diabetes mellitus, *CAD* coronary artery disease, *NT-proBNP* n-terminal pro B-type natriuretic peptide, *Hs Troponin T* high sensitivity troponin T, *GFR* glomerular filtration rate

Bold refers to significant p-values, defined as *p*<0.05

**Fig. 1:**
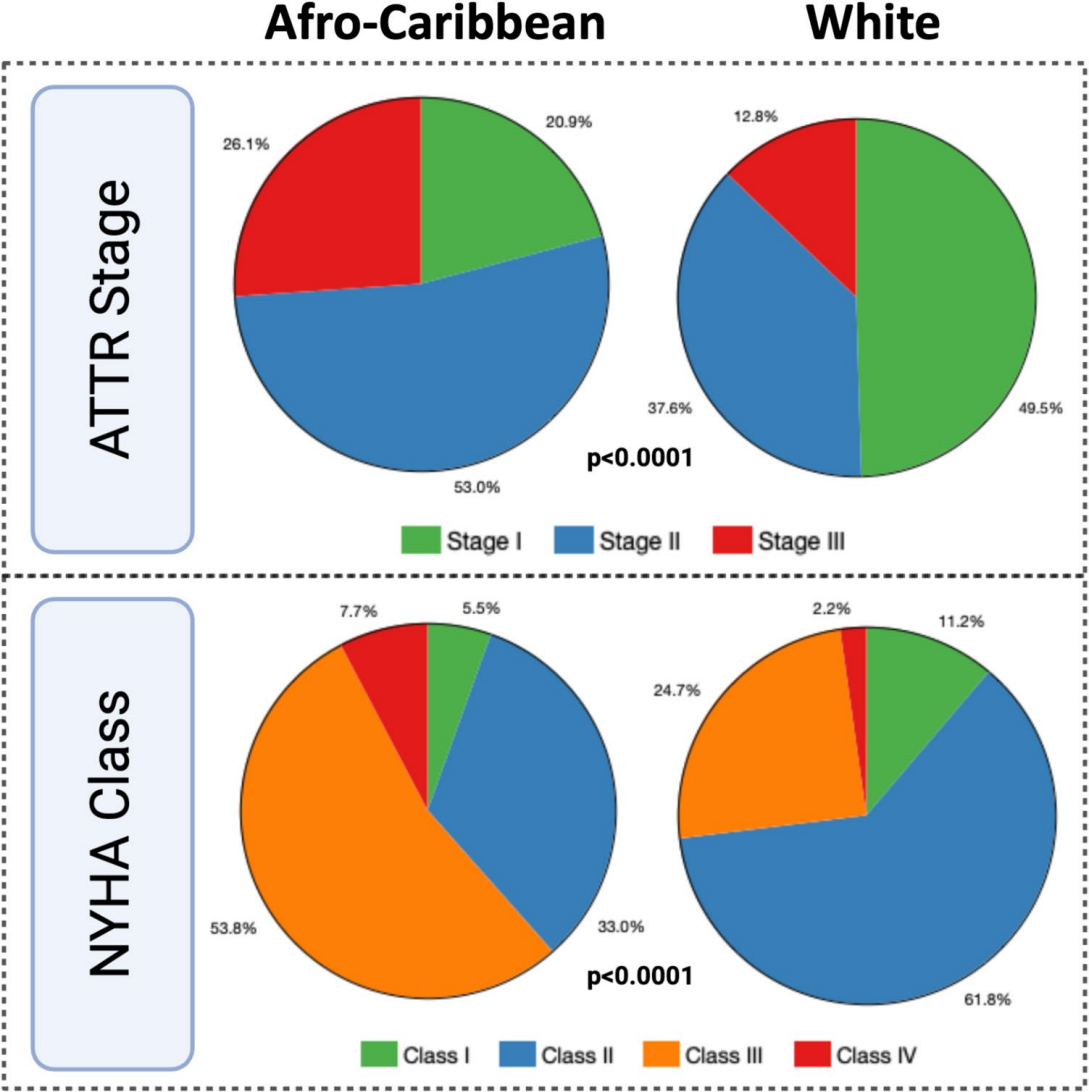
Comparison of UK ATTR-CA stage and NYHA Class based on race at the time of diagnosis in African American vs White patients (ATTR, transthyretin; CA, cardiac amyloidosis; NYHA, New York Heart Association; UK, United Kingdom)

Comparison of clinical parameters between AC and white patients is displayed Table 1. The AC group had a higher prevalence of hypertension (79% vs 58%, p < 0.001) and diabetes (35% vs 20%, p = 0.01), while CAD was more prevalent in whites (38% vs 23%, p = 0.02). AC patients were found to present with a significantly higher NYHA class at the time of diagnosis (III and IV), while whites often presented with a lower NYHA class (I and II) (Fig. 1). The AC group had a higher prevalence of pathogenic gene mutations for ATTR (74% vs 18%, p < 0.0001). Among the AC patients with genetic mutations, 95% were Val142Ile and 5% other. Among the white patients with genetic mutations, 38% were Thr60 Ala, 25% Val142Ile, 13% Val30Met, and 24% other. Compared to white patients, AC patients were less likely to receive treatment with ATTR stabilizer or small inhibitor ribonucleic acid inhibitors during the study period (siRNA) (Table 1).

### Lab biomarkers

A comparison of serum biomarkers between AC and white patients is also displayed in Table 1. The median levels of NT-proBNP and troponin were elevated within the entire group, measuring 2462 pg/ml and 67 ng/dl, respectively, while eGFR was reduced at 59 ml/min/1.73 m^2^. AC group exhibited significantly higher levels of NT-proBNP (p < 0.001) and a higher level of high-sensitivity troponin (p = 0.01) at the time of diagnosis. Additionally, AC individuals were found to have a lower average eGFR compared to whites (p = 0.002, Table 1). AC patients also had significantly higher UK ATTR-CA disease stages at the time of diagnosis (Fig. 1).

### Imaging parameters

A comparison of CMR parameters between AC and white patients is displayed in Table 2. AC patients had lower LVEF (40 ± 13% vs 46 ± 11%, p = 0.001) and RVEF (39 ± 11% vs 46 ± 11%, p = 0.0002) compared to whites. AC patients were also found to have a smaller maximal wall thickness [18 (IQR 16–20) vs 20 (IQR 17–22) mm, p = 0.003], higher extracellular volume [58% (IQR 49–69%) vs 50% (45–59%), p = 0.001), and a higher rate of pericardial effusion (32% vs 17%, p = 0.03). Other CMR measurements and LGE distribution were similar between the two groups.

**Table 2:** Comparison of CMR parameters between AC and white patients

	Entire Cohort (n = 231)	AC (n = 114)	White (n = 117)	P value
Indexed LV Mass (gm/m^2^)	98 (82–177)	98 (80–112)	97 (84–122)	0.30
Indexed LVEDV (mL/m^2^)	84 (73–99)	81 (71–102)	87 (76–97)	0.49
Indexed LVESV (mL/m^2^)	48 (39–61)	51 (39–69)	46 (38–57)	0.08
LVEF	43 ± 13%	40 ± 13%	46 ± 11%	**0.001**
Indexed RVEDV (mL/m^2^)	89 (72–106)	93 (74–110)	85 (70–101)	0.11
Indexed RVESV (mL/m^2^)	52 (38–68)	59 (41–73)	46 (34–61)	**0.006**
RVEF	42 ± 12%	39 ± 11%	46 ± 11%	**0.0002**
Indexed LA volume (mL/m^2^)	58 ± 17	57 ± 16	59 ± 19	0.49
Max wall thickness (mm)	18 (17–21)	18 (16–20)	20 (17–22)	**0.003**
ECV	54% (46–62%)	58% (49–69%)	50 (45–59%)	**0.001**
Pericardial effusion n(%)	41 (25%)	28 (32%)	13 (17%)	**0.03**
LGE Pattern				0.76
Absent	0 (0%)	0 (0%)	0 (0%)	
Atypical	31 (16%)	17 (16%)	14 (15%)	
Typical	166 (84%)	86 (84%)	80 (85%)	

*AC* Afro-Caribbean, *EDV* end diastolic volume, *EF* ejection fraction, *ESV* end systolic volume, *LA* left atrium, *LGE* late gadolinium enhancement, *LV* left ventricle, *RV* right ventricle

Bold refers to significant p-values, defined as *p*<0.05

### Clinical outcomes

At a median follow-up time of 365 (97–879) days, 44% of patients had experienced the primary endpoint of death or HFH and 34% had died. Between the two groups, AC had higher mortality rates (46% vs 23%, p < 0.001) and were more likely to be hospitalized due to heart failure (65% vs 22%, p < 0.0001). The univariate associations between clinical parameters and the primary endpoint of death or HFH are displayed in Table 3. AC race carried the strongest association with the primary endpoint with a HR 2.85 (1.92–4.23), p < 0.0001. Female sex, genotype, hypertension, and diabetes were also significantly associated with the primary endpoint.

**Table 3:** Cox regression analysis depicting the relationship between clinical parameters and the primary endpoint of death or heart failure hospitalization

Parameter	Hazard ratio	P-value
AC race	2.85 (1.92–4.23)	** < 0.0001**
Female sex	1.77 (1.15–2.71)	**0.009**
Mutant genotype	1.59 (1.04–2.43)	**0.03**
Hypertension	1.77 (1.17–2.69)	**0.007**
Diabetes	1.75 (1.20–2.56)	**0.004**
Coronary artery disease	1.28 (0.85–1.91)	0.24

Bold refers to significant p-values, defined as *p*<0.05

The relationship between clinical parameters and the secondary individual endpoints is displayed in Supplementary Tables 1, 2. AC race was associated with both death (p = 0.01) and HFH (p < 0.0001) individually. The presence of a pathogenic ATTR mutations (HR: 1.73, CI 1.08–2.77, p = 0.02), hypertension (HR: 1.78, CI 1.12–2.94, p = 0.002), and diabetes (HR: 1.95, CI 1.29–2.94, p = 0.02) were significantly associated with HFH but not with death.

**Fig. 2:**
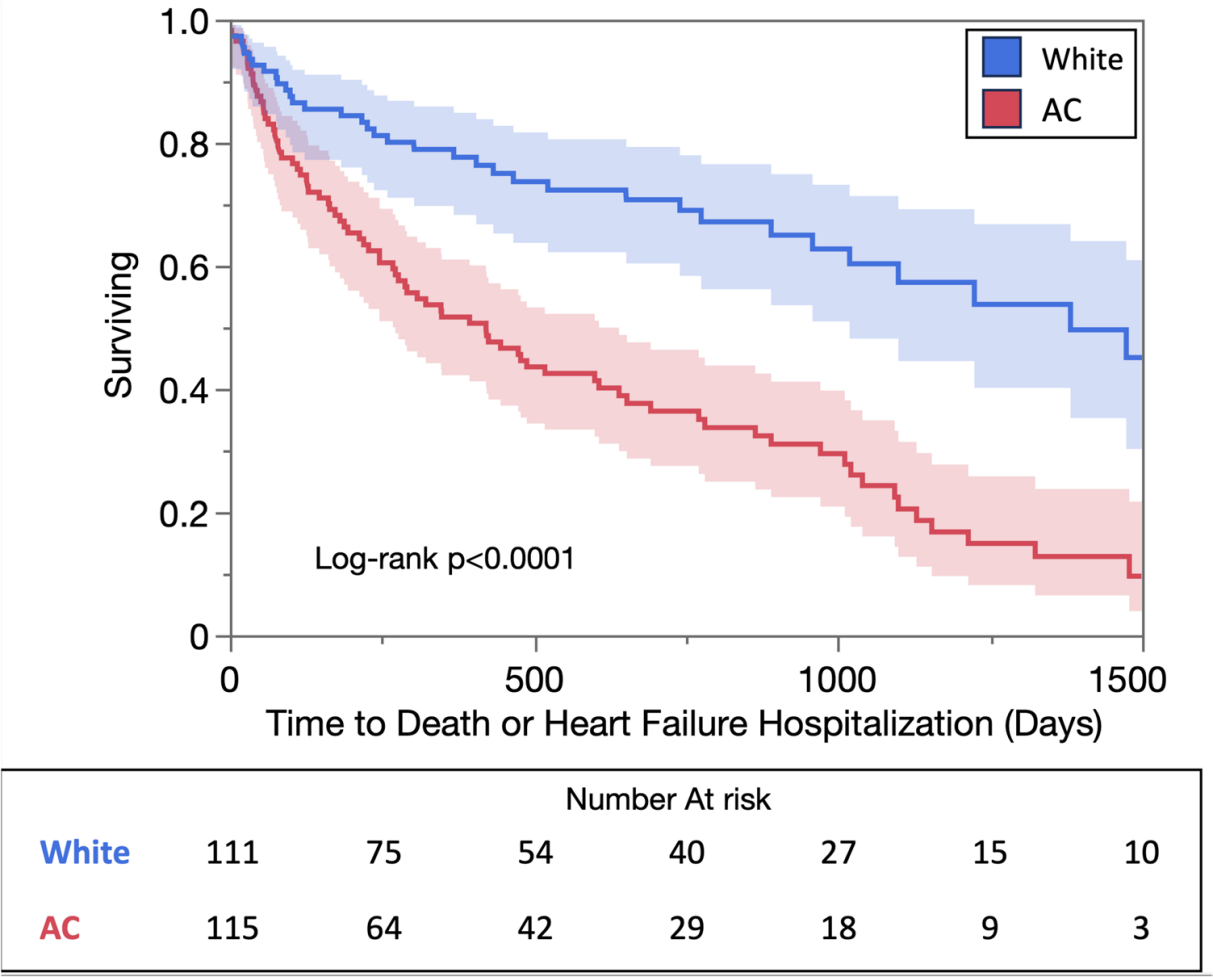
Kaplan–Meier analysis depicting the relationship between race and the primary endpoint of death or heart failure hospitalization

Kaplan–Meier analysis depicting the unadjusted relationship between race and the primary and secondary endpoints is displayed in Fig. 2. AC race was significantly associated with the primary endpoint of death or HFH (chi-square 29.3, p < 0.0001). AC race was also significantly associated with both death (chi-square 6.8, p = 0.009) and HFH (chi-square 36.6, p < 0.0001) individually (Supplementary Fig. 1).

### Multivariable analysis

**Fig. 3:**
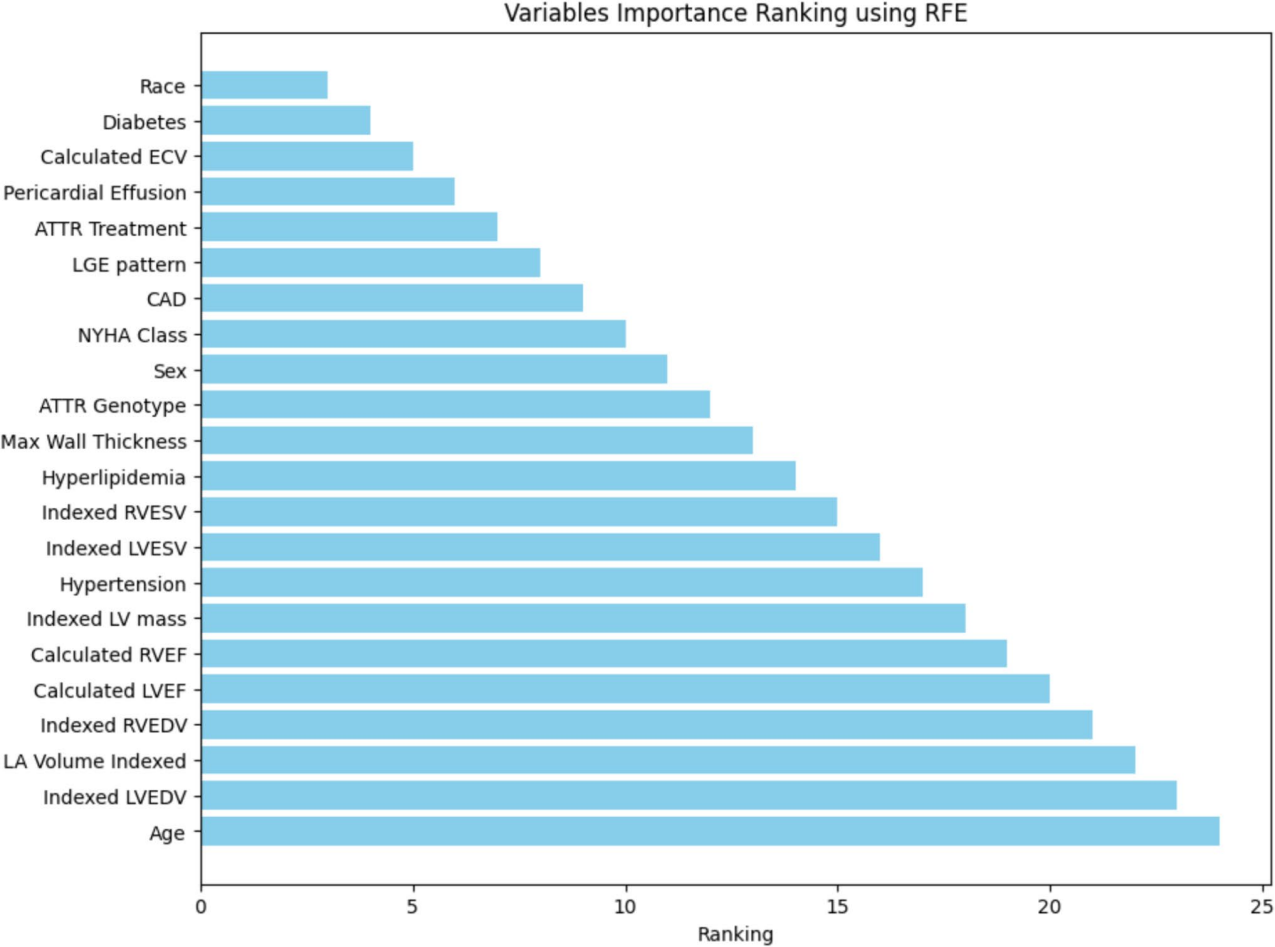
Variables importance ranking for predicting the primary endpoint of death or heart failure hospitalization as determined by the Recursive Feature Elimination (RFE) method. Variables with lower rank are considered more important (race being the most important and age the least important)

After adjusting for significant covariates, including sex, hypertension, ATTR genotype, CAD, diabetes and the Mayo Clinic staging available in our dataset, AC race remained significantly associated with the primary endpoint (HR: 1.68, 95% CI 1.1–2.6, p = 0.02). Furthermore, time of diagnosis was not significantly different between races (p = 0.58), and time of diagnosis did not significantly change the association of AC with clinical outcomes in multivariable analysis (p = 0.12). Fine and Gray proportional sub-distribution hazards model confirmed that AC race was associated with HFH after adjusting for significant covariates, with an estimated sub-distribution HR of 2.60 (95% CI 1.44–4.69, p = 0.002). In exploratory analysis controlling for genotype, AC race remained significantly associated with MACE in those with pathogenic gene mutations (chi-square 9.2, log-rank p < 0.003) with a trend towards significance in wildtype patients (Supplementary Fig. 2 A, B). Among those with wild type genotype, there was a non-significant trend towards worse survival amongst AC patients. The results of multivariable RFE are displayed in Fig. 3. AC race was the most important parameter for predicting time to the incidence of the primary endpoint of death or HFH. Race outperformed all the analyzed clinical and CMR-based parameters for the incidence of the primary endpoint.

## Discussion

In this large multicenter study, we hypothesized that AC race would be associated with worse disease-related outcomes in ATTR-CA. Indeed, we found that AC race was associated with: (1) greater symptoms, (2) more advanced disease burden at diagnosis, and (3) lower rates of treatment with ATTR-CA therapies. Additionally, AC race was the most important predictor of death or HFH within the cohort in multivariable analysis.

AC patients exhibited greater derangements in serum biomarkers—including NT-proBNP, troponin, and renal function—compared with white patients at the time of diagnosis. These parameters are established staging biomarkers and are strongly linked to prognosis in this disorder [17, 31]. Similarly, AC patients had more advanced UK ATTR-CA disease stages and NYHA classification at the time of diagnosis. Our findings align with previous large, multicenter work by Cavalier et al*.,* which also showed increased biomarker derangement and more advanced heart failure staging at the time of ATTR-CA diagnosis in AC patients (reference Cavalier here). It is particularly interesting that there were no significant intergroup differences in age at the time of diagnosis despite a higher prevalence of Val142Ile mutations—a mutation which is associated with earlier age of disease onset—in the AC cohort [32]. In our study, AC patients exhibited a more advanced cardiac phenotype at the time of diagnosis, as evidenced by worse biventricular ejection fraction and higher ECV. ECV is a robust, non-invasive marker, which has consistently been shown to correlate with both amyloid burden and prognosis [33–36]. To our knowledge, our study is the first to demonstrate race-based differences in cardiac structure and function at the time of ATTR-CA diagnosis using CMR.

Overall, these findings suggest substantial delays in diagnosis of AC patients with respect to their white counterparts, possibly resulting in more advanced illness and symptoms at the time of CA detection. Social determinants of health, such as access to care and medical insurance, as well as poor trust of the medical system, may play a significant role in these race-based differences [37, 38]. Clinician-related factors which may contribute to delayed diagnosis are failure to suspect ATTR-CA and potential implicit biases, which have been shown to impact clinician decision-making regarding the pursuit of appropriate diagnostic testing in other cardiovascular disorders [9, 39]. Further research is needed to better understand the specific mechanisms, which may contribute to delayed diagnosis in ATTR-CA in AC patients. AC also had a higher prevalence of comorbidities, such as diabetes and hypertension, which were also significantly associated with HFH, emphasizing the multifactorial nature of healthcare disparities in this population [37]. Diagnosis and appropriate management of these comorbid conditions may also be negatively impacted by social factors, such as access to care, medication affordability, psychological stress, and social support, which may further compound the disproportionate cardiovascular disease burden in AC patients [37].

These race-based differences in illness stage at the time of diagnosis are particularly concerning given the growing recognition that ATTR-CA therapies may be less effective when treatment is delayed further into the disease course. In sub-analysis from the initial landmark ATTR-ACT study, there was a lack of demonstrated benefit amongst those who were NYHA Class IV at the time of treatment initiation [1]. In longer term open-label follow up of the same trial, those in whom tafamidis was initiated later, at the conclusion of the initial study, experienced worse outcomes compared to those randomized to the drug at the onset of the trial, irrespective of age and NYHA Class at the time of randomization [40, 41]. The availability of therapy for ATTR-CA underscores the critical importance of early diagnosis and initiation of appropriate treatment, particularly in populations with heightened vulnerability, such as AC.

Additionally concerning was the finding that AC race was associated with lower rates of treatment with targeted ATTR-CA therapies, such as transthyretin stabilizers and/or siRNA. It is unknown whether this was influenced by patients’ clinical or social factors, or clinician/systems-driven barriers. Further research is needed to better understand the specific mechanisms underlying race-based differences in ATTR-CA care.

In our study, AC race was also associated with worse outcomes in ATTR-CA. Even after accounting for clinical parameters such as ATTR genotype and CMR-based parameters, race remained the single most important parameter for predicting the primary endpoint of death or HFH. AC race was also significantly associated with death and HFH individually. Our findings align with recent work by Khedraki et al. in which they demonstrated increased incidence of death and need for advanced therapies in AC versus white patients [42].

Similarly, race has been associated with worse outcomes across the spectrum of heart failure. An analysis using the multiple causes of death files from the CDC and Prevention’s Wide-Ranging Online Data for Epidemiologic Research highlighted that AC men had a 1.16-fold versus 1.43-fold higher age-adjusted HF-related CVD death rate, compared with white men, and AC women had a 1.35-fold versus 1.54-fold higher age-adjusted HF-related CVD death rate compared with white women in 1999 versus 2017 (p < 0.05) [11]. Similarly, previous studies have reported a 2.5-fold higher HFH rate amongst AC heart failure patients compared to whites [43]. Readmission rates at 30 days [44] and one year are also higher [45]. AC race is also associated with worse outcomes in patients with advanced heart failure undergoing heart transplantation and left ventricular assist device implantation [46, 47].

Ultimately, we hypothesize that delays in diagnosis may drive undertreatment and worse outcomes in ATTR-CA. Our findings highlight the need for prompt exploration of potential factors such as social determinants of health, comorbidities, diagnostic delays, and undertreatment which may contribute to healthcare disparities in this disorder. This includes a careful examination of socioeconomic factors, healthcare utilization patterns, treatment adherence, and implicit bias on treatment and outcomes in ATTR-CA. These alarming findings also underscore the urgency to implement targeted interventions to address the underlying healthcare disparities and improve outcomes in AC patients with ATTR-CA. Future investigations should focus on elucidating the specific mechanisms driving these potential disparities, paving the way for more effective and equitable healthcare strategies in the management of ATTR-CM.

## Limitations

Our study has several limitations. The retrospective design and relatively small sample size constrained our ability to control for all potential confounders such as genotypes. Genotypes such as Val142Ile are known to result in earlier onset and appear to have a more aggressive cardiac phenotype compared to wild-type counterparts [48, 49]. It is important to note that although outperformed ATTR genotype for prediction of MACE in RFE analysis, our study underpowered to fully control for genotype. Given the small percentage of white Val142Ile positive patients and AC wild type patients, larger cohort sizes would be needed for this analysis. Still, in our exploratory analysis, race remained significantly associated with MACE in those with pathogenic gene mutations with a trend towards significance in wild type patients. This along with the RFE findings suggest that genotype alone may not account for race-based differences in outcomes. Further studies are needed to better elucidate the interaction between race, genotype, and outcomes in ATTR-CA. Additionally, the study took place over a long period of time, during which the availability of therapies may have changed; however, there was no significant difference in date of diagnosis between races, suggesting that this is unlikely to have impacted the key outcomes. Information regarding precise dates of treatment initiation were not available within the SCMR Registry and was therefore not uniformly available across all sites; therefore, we are unable to adjust for timing of treatment initiation—and its potential confounding effect on outcomes—relative to MACE in this cohort. This represents an important area for future study. Additionally, including patients from different institutions using various MRI scanners introduces variability in tissue mapping parameters. We therefore chose to rely on ECV, rather than T1 and T2 mapping, which has shown more consistency across various scanners and field strengths [50]. Furthermore, while our study examined the impact of race on adverse disease markers and outcomes, we did not explore specific drivers of these outcomes, such as access to care, medical insurance, trust in the medical field, and implicit bias. Lastly, race was derived from the EMR and may not truly represent patient self-identification.

## Conclusion

In patients with ATTR-CA, AC race is associated with more advanced disease stage at the time of diagnosis, reduced treatment with life prolonging therapies, and worse outcomes in ATTR-CA. Further studies are needed to understand the specific drivers of poor outcomes in AC patients with ATTR-CA with the aim of ultimately developing targeted strategies aimed to address healthcare disparities in this patient population.

## Supplementary Information

Below is the link to the electronic supplementary material.

Supplementary file1 (DOCX 359 KB)

## Data Availability

No datasets were generated or analysed during the current study.
