# Supplementary material for: Disparities in diagnosis and outcomes in American patients with transthyretin cardiac amyloidosis
Source: Int J Cardiovasc Imaging. 2025 Jun 5;41(8):1487–96. doi: 10.1007/s10554-025-03436-4 (PMC12397186; doi:10.1007/s10554-025-03436-4)
Supplement: Supplementary file 1 — Supplementary file1 (DOCX 359 KB) [file 10554_2025_3436_MOESM1_ESM.docx]

**Supplementary Tables:**

| **Supplementary Table 1:** Cox regression depicting the relationship between clinical parameters and death. | | |
| --- | --- | --- |
| **Parameter** | **Hazard Ratio** | **P-value** |
| **Clinical Parameters** |  |  |
| AC Race | 1.85 (1.16-2.96) | **0.01** |
| Female Sex | 1.36 (0.78-2.37) | 0.27 |
| Mutant Genotype | 1.18 (0.70-2.02) | 0.53 |
| Hypertension | 1.50 (0.91-2.47) | 0.11 |
| Diabetes | 1.48 (0.93-2.35) | 0.10 |
| Coronary Artery Disease | 1.05 (0.66-1.68) | 0.83 |

| **Supplementary Table 2:** Cox regression depicting the relationship between clinical parameters and heart failure hospitalization. | | |
| --- | --- | --- |
| **Parameter** | **Hazard Ratio** | **P-value** |
| **Clinical Parameters** |  |  |
| AC Race | 3.88 (2.42-6.22) | **<0.0001** |
| Female Sex | 1.49 (0.90-2.44) | 0.12 |
| Mutant Genotype | 1.73 (1.08-2.77) | **0.02** |
| Hypertension | 1.78 (1.12-2.84) | **0.02** |
| Diabetes | 1.95 (1.29-2.94) | **0.002** |
| Coronary Artery Disease | 1.31 (0.84-2.06) | 0.24 |

**Supplementary Figure 1:** Kaplan-Meier analysis depicting the relationship between race and both A) death and B) heart failure hospitalization.

**Supplementary Figure 2:** Kaplan-Meier analysis depicting the relationship between race and the primary endpoint of death or heart failure hospitalization in A) genotype positive and B) wild type ATTR-CA.
